# Supplementary material for: Explainable AI decision support improves accuracy during telehealth strep throat screening
Source: Commun Med (Lond). 2024 Jul 24;4:149. doi: 10.1038/s43856-024-00568-x (PMC11269612; doi:10.1038/s43856-024-00568-x)
Supplement: Supplementary file 2 — Supplementary material [file 43856_2024_568_MOESM2_ESM.pdf]

# Supplementary information

## Supplementary Method

Trust-related statements presented at each case:[1]

- I am confident about the system's capability to predict strep throat
- The system works reliably
- I trust the system

Subjective understanding statements presented at each case:

- I was able to understand why the system resulted/predicted the way it did[1]
- Although I may not know exactly how the system works, I know how to use it to make decisions about the problem[2]
- The system description lets me judge when I should/shouldn't trust the system[3]

In the statements, system can be replaced by Centor Score or AI.

## Supplementary Figures

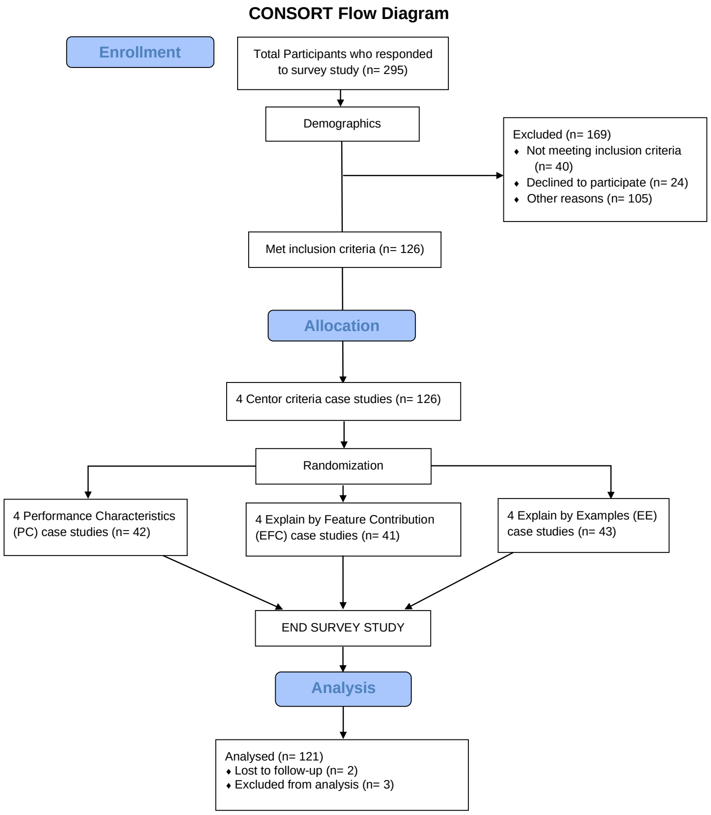


Figure 1: CONSORT Flow Diagram for participant enrollment in the study.

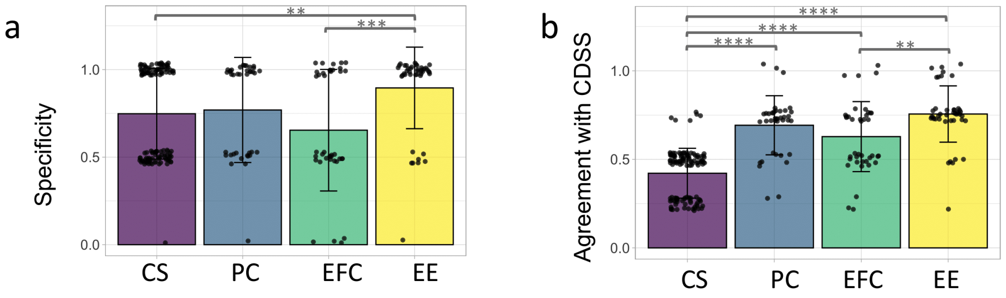


Figure 2: Bar plots of measures for different types of support information in the CDSS. CS corresponds to Centor Score, PC to Performance Characteristics, EFC to Explain by Feature Contribution, and EE to Explain by Examples. a) Specificity (N=242 observations) and b) Agreement with the CDSS diagnostic advice (N=242 observations). The error bars shown in the plots represent the standard deviation and significant results are emphasized with gray lines. *p≤0.05, **p≤0.01, ***p≤0.001, ****p≤0.0001

## Supplementary Tables

Table 1: Valid participants ($n$ = 121) demographics and additional characteristics.

| Categorical Variables | Group | Count | % of total |
|---|---|---|---|
| **Age (years)** | 18-29 | 8 | 6.6 |
| | 30-39 | 64 | 52.9 |
| | 40-49 | 25 | 20.7 |
| | 50-59 | 13 | 10.7 |
| | 60-69 | 8 | 6.6 |
| | 70+ | 3 | 2.5 |
| **Sex** | Female | 90 | 74.4 |
| | Male | 31 | 25.6 |
| | Intersex | 0 | 0 |
| | Prefer not to answer | 0 | 0 |
| **Type of provider** | Physician | 48 | 39.7 |
| | NP | 13 | 10.7 |
| | PA | 60 | 49.6 |
| **Experience (years)** | 1-3 | 22 | 18.2 |
| | 4-10 | 42 | 34.7 |
| | 11-15 | 23 | 19.0 |
| | 16-20 | 10 | 8.3 |
| | 20+ | 24 | 19.8 |
| **Clinical Setting** | Primary Care | 77 | 63.6 |
| | Urgent Care | 51 | 42.1 |
| | Emergency Medicine | 10 | 8.3 |
| | Fully virtual/remote | 27 | 22.3 |
| NumericalVariables | Mean (SD) | | |
| **Percent Telehealth visits** | 40.3 (35.3) | | |

Table 2: Descriptive statistics of specificity, positive and negative predictive values for each type of CDSS. The values resported correspond to mean (standard deviation).

| Metric | Centor Score | Performance Characteristics | Explain by Feature Contribution | Explain by Examples |
|---|---|---|---|---|
| Positive Predictive Value | 0.12 (0.27) | 0.81 (0.24) | 0.71 (0.26) | 0.91 (0.19) |
| Negative Predictive Value | 0.45 (0.10) | 0.68 (0.24) | 0.57 (0.28) | 0.71 (0.20) |

Table 3: Linear models for additional performance outcomes. Centor score was the reference level. Adjusted $R^2 = 0.668$ for the PPV model and Adjusted $R^2 = 0.275$ for the NPV model.

| Measure | Predictors | Estimate | Standard Error | t | p-value |
|---|---|---|---|---|---|
| Positive predictive value | Intercept | 0.18 | 0.04 | 4.17 | $4.3 \times 10^{-5}$ |
| | PC | 0.69 | 0.05 | 15.08 | $< 2.0 \times 10^{-16}$ |
| | EFC | 0.59 | 0.05 | 12.96 | $< 2.0 \times 10^{-16}$ |
| | EE | 0.79 | 0.04 | 17.85 | $< 2.0 \times 10^{-16}$ |
| Negative predictive value | Intercept | 0.44 | 0.03 | 13.81 | $< 2.0 \times 10^{-16}$ |
| | PC | 0.22 | 0.03 | 6.70 | $1.6 \times 10^{-10}$ |
| | EFC | 0.12 | 0.03 | 3.69 | $2.8 \times 10^{-4}$ |
| | EE | 0.27 | 0.03 | 8.32 | $7.5 \times 10^{-15}$ |

## References


[1] Moritz Körber. Theoretical considerations and development of a questionnaire to measure trust in automation. In *Proceedings of the 20th Congress of the International Ergonomics Association (IEA 2018) Volume VI: Transport Ergonomics and Human Factors (TEHF), Aerospace Human Factors and Ergonomics 20*, pages 13–30. Springer, 2019.

[2] Maria Madsen and Shirley Gregor. Measuring human-computer trust. In *11th australasian conference on information systems*, volume 53, pages 6–8. Citeseer, 2000.

[3] Robert R Hoffman, Shane T Mueller, Gary Klein, and Jordan Litman. Metrics for explainable ai: Challenges and prospects. *arXiv preprint arXiv:1812.04608*, 2018.
