# Supplementary material for: Explainable AI decision support improves accuracy during telehealth strep throat screening
Source: Commun Med (Lond). 2024 Jul 24;4:149. doi: 10.1038/s43856-024-00568-x (PMC11269612; doi:10.1038/s43856-024-00568-x)
Supplement: Supplementary file 3 — Description of Additional Supplementary Files [file 43856_2024_568_MOESM3_ESM.pdf]

Description of Additional Supplementary Files

File name: Supplementary Data 1

File Description: files: survey_responses.csv, objective_measures.csv, analysis_code.R
